# The mediating role of the venules between smoking and ischemic stroke

**DOI:** 10.1007/s10654-018-0436-2

**Published:** 2018-09-04

**Authors:** Unal Mutlu, Sonja A. Swanson, Caroline C. W. Klaver, Albert Hofman, Peter J. Koudstaal, Muhammad Arfan Ikram, Muhammad Kamran Ikram

**Affiliations:** 1 Department of Epidemiology, Erasmus Medical Center, PO Box 2040, 3000 CA Rotterdam, The Netherlands; 2 Department of Ophthalmology, Erasmus Medical Center, Rotterdam, The Netherlands; 3 Department of Epidemiology, Harvard T.H. Chan School of Public Health, Harvard University, Boston, MA USA; 4 Department of Neurology, Erasmus Medical Center, Rotterdam, The Netherlands; 5 Department of Radiology, Erasmus Medical Center, Rotterdam, The Netherlands

**Keywords:** Mediation, Interaction, Smoking, Venules, Stroke, Population-based study

## Abstract

A potential mechanism by which smoking affects ischemic stroke is through wider venules, but this mediating role of wider venules has never been quantified. Here, we aimed to estimate to what extent the effect of smoking on ischemic stroke is possibly mediated by the venules via the recently developed four-way effect decomposition. This study was part of a population-based study including 9109 stroke-free persons participated in the study in 1990, 2004, or 2006 (mean age: 63.7 years; 58% women). Smoking behavior (smoking versus non-smoking) was identified by interview. Retinal venular calibers were measured semi-automatically on retinal photographs. Incident strokes were assessed until January 2016. A regression-based approach was used with venular calibers as mediator to decompose the total effect of smoking compared to non-smoking into four components: controlled direct effect (neither mediation nor interaction), pure indirect effect (mediation only), reference interaction effect (interaction only) and mediated interaction effect (both mediation and interaction). During a mean follow-up of 12.5 years, 665 persons suffered an ischemic stroke. Smoking increased the risk of developing ischemic stroke compared to non-smoking with an excess risk of 0.41 (95% confidence interval 0.10; 0.67). With retinal venules as a potential mediator, the excess relative risk could be decomposed into 77% controlled direct effect, 4% mediation only, 4% interaction only, and 15% mediated interaction. To conclude, in the pathophysiology of ischemic stroke, the effect of smoking on ischemic stroke may partly explained by changes in the venules, where there is both pure mediation and mediated interaction.

## Introduction

Stroke is a major cause of mortality and disability among elderly people worldwide, and its burden is expected to increase even further [1]. As it is well-established that smoking increases the risk of ischemic stroke, mechanisms by which this occurs have been broadly investigated in recent decades. One possible mechanism by which smoking may lead to ischemic stroke is through changes in the cerebral venules [2]. Existing evidence shows that damage to the venules plays an important role in the development of ischemic stroke [3]. While the association between smoking and wider venules is firmly established by several studies, residual confounding cannot be fully excluded, and the exact underlying mechanisms need to be explored. Of all stroke, it has been estimated that about 20% is caused by small vessel disease [4, 5]. It has been shown that smoking is associated with wider venules, implicating that changes in cerebral venules mediate the effect of smoking on ischemic stroke [2]. Nevertheless, it remains unclear whether the effects of smoking on ischemic stroke are mediated through wider venules, whether there is interaction between smoking and wider venules, or whether a combination of mediation and interaction could be occurring. While studies have shown an interaction and mediation between smoking and major cardiovascular risk factors for stroke such as hypertension, diabetes mellitus, and high serum lipid levels [6–8], its role with wider venules remains unclear.

In epidemiologic studies, questions pertaining to mediation have been traditionally tackled with methods that assume no exposure-mediator interaction. Recent advances in the conceptual framework of causal mediation allow estimating the direct and indirect effect even in the presence of an exposure-mediator interaction [9]. Moreover, these advances allow further effect decomposition to explore both mediation and interaction simultaneously i.e. to decompose the exposure’s effect on the outcome into components related to mediation only, interaction only, both, or neither (formal definitions are provided below) [10]. When free of bias, this approach provides insights into relevant processes in the pathways under study, and enables researchers to better understand biological mechanisms. Thus far, the four-way decomposition method has not been extensively used in clinical research. Application of this counterfactual approach of causal mediation analysis in clinical research may provide insights into the pathophysiology of clinical outcomes. Moreover, in terms of future interventions, this approach may aid the clinician in identifying the best target for treatment, or even show why a certain treatment does not work.

In this study, we aimed to understand the role of the venules in the effect of smoking on ischemic stroke by applying the four-way decomposition method.

## Methods

### Setting and study population

**Fig. 1:**
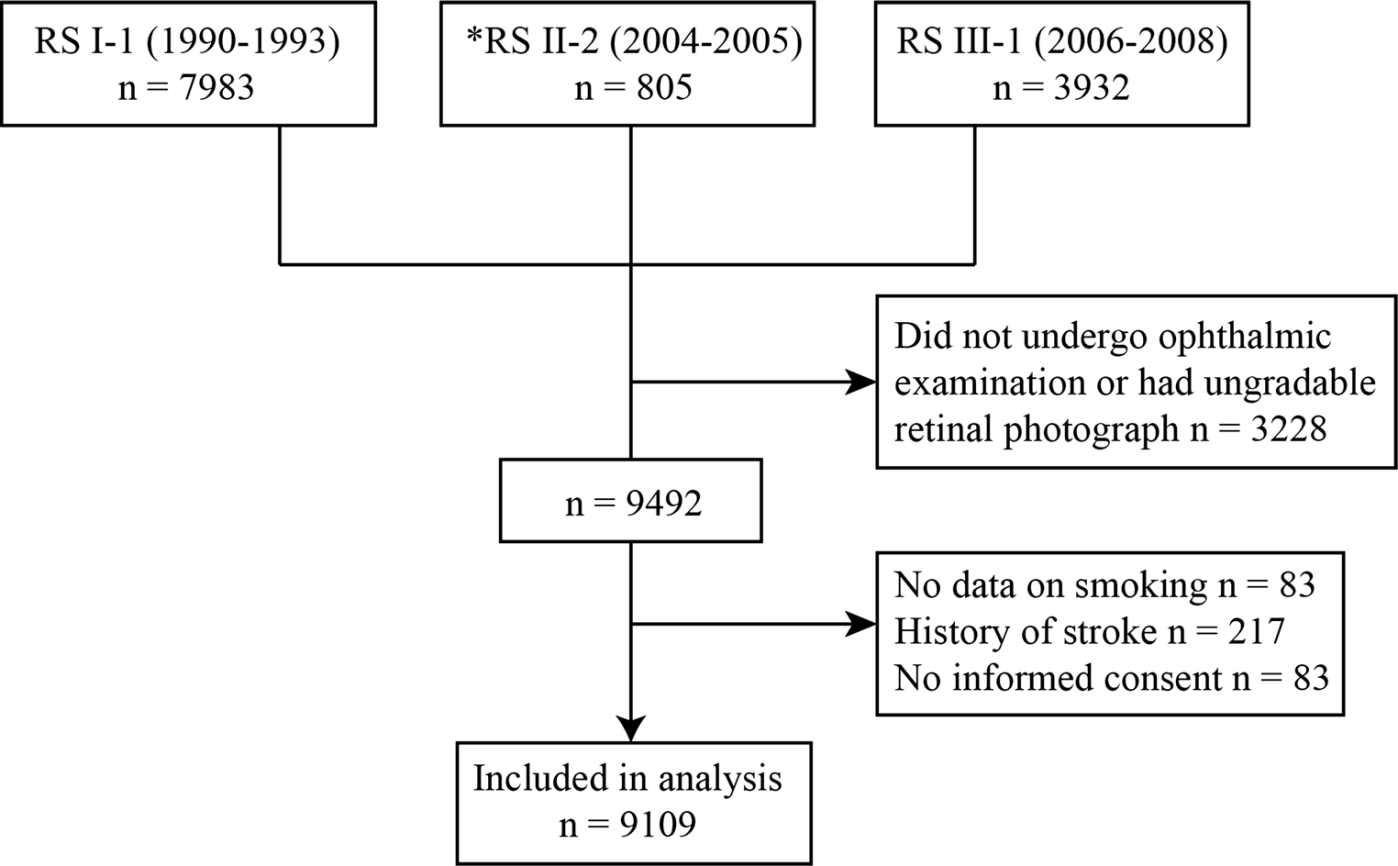
Flow diagram of the study. *Retinal images of 805 persons of the total 2506 participants were randomly selected for retinal caliber measurement

This study is based on the Rotterdam Study (RS), a large prospective population-based cohort study in the Netherlands that investigates causes and consequences of chronic diseases in the general population [11]. All inhabitants of the Ommoord district in the city Rotterdam, aged 55 years or older, were invited to the study in 1990 (RS-I). In 2000 those who had become 55 years of age or moved into the study district were invited (RS-II). In 2006 a further extension of the cohort was initiated and inhabitants aged 45 years or older were invited (RS-III). Home interviews including assessment of smoking, and physical examinations take place every 3–4 years. Retinal vascular calibers were measured at the baseline visit of RS-I (RS-I-1), in a random sample of the second visit of RS-II (RS-II-2), and at the baseline visit of RS-III (RS-III-1), see Fig. 1. We considered the date at which smoking assessment was done in RS-I-1, RS-II-2 and RS-III-1 as our baseline. We excluded persons without data on smoking, without gradable retinal photographs, persons with a history of stroke at baseline, and persons who did not give permission to monitor for future disease event. The Rotterdam Study has been approved by the Medical Ethics Committee of the Erasmus MC and by the Ministry of Health, Welfare and Sport of the Netherlands, implementing the “Population Studies Act: Rotterdam Study” (Wet Bevolkingsonderzoek: ERGO). All participants provided written informed consent to participate in the study and to obtain information from their treating physicians.

### Assessment of smoking

Information on smoking behavior was obtained using a computerized questionnaire during the home visits. Participants were classified as current smokers, or non-smokers (including both former or never smokers). Current smokers were participants who answered yes to the question: “are you currently smoking?” Former smokers were participants who answered no to this question and answered positively to the question: “are you a former smoker?”

### Assessment of the venules

Participants underwent a full eye examination at each subcohort’s baseline (1990, 2004, and 2006) including simultaneous stereoscopic retinal color photography of the optic disc (20° field, Topcon Optical Company, Tokyo, Japan) of both eyes after pharmacological mydriasis. For each participant, the image of one eye with the best quality was analyzed with the Retinal Vessel Measurement System (Retinal Analysis, Optimate, WI; Department of Ophthalmology & Visual Science, University of Wisconsin-Madison). For each participant one summary value was calculated for the arteriolar diameters and one for the venular diameters (in μm) of the blood column after correction for differences in magnification due to refractive status of the eye, enabling us to use the separate arteriolar and venular diameter sum values [12, 13]. We verified in a random sub-sample of 100 participants in RS-I that individual measurements in the left and right eye were similar. Measurements were performed by total four trained raters, masked for participant characteristics. Pearson’s correlation coefficient for interrater agreement were for arteriolar diameters 0.67–0.87, and for venular diameters 0.91–0.94. For intrarater agreement the correlation coefficients were 0.65–0.88 for arteriolar diameters, and 0.82–0.95 for venular diameters.

### Assessment of stroke

History of stroke was assessed using home interviews and confirmed by reviewing medical records. Participants were continuously followed up for stroke through digital linkage of general practitioners’ files with the study database [14]. Furthermore, nursing home physicians’ files and files from general practitioners of participants who moved out of the district were checked on a regular basis. Hospital discharge letters and information from general practitioners was collected for all potential strokes. Research physicians reviewed the information and an experienced neurologist verified the strokes. Strokes were further classified into ischemic or hemorrhagic based on neuroimaging reports. Subarachnoid hemorrhages were excluded. Infarcts that turned hemorrhagic were classified as ischemic stroke. If neuroimaging was lacking, a stroke was classified as unspecified. Follow-up was completed until 1 January 2016. Participants suffering stroke at any point in follow-up were dichotomized as yes/no.

### Assessment of covariates

We considered the “smoking/ischemic stroke” relation, and “wider venules/ischemic stroke” relation to be confounded by the same set of measured persons characteristics. All confounders were assessed at the same visit period when smoking was assessed and when retinal photographs were obtained. Blood pressure was measured twice in sitting position at the right brachial artery with a random-zero sphygmomanometer, and the average of two readings was used for analysis. Body mass index was computed as weight (kg) divided by height squared (m^2^). Serum total and high-density lipoprotein cholesterol concentrations were determined by means of an automated enzymatic procedure. White blood cell count was assessed in citrate plasma using a Coulter counter T540 (Coulter electronics, Luton, England). Diabetes mellitus was considered present if participants reported use of antidiabetic medication or when non-fasting serum glucose level was greater than 11 mmol/l, or when fasting glucose level in serum was greater than 7 mmol/l. Alcohol consumption was calculated as amount of alcohol in g/day. Atherosclerotic plaques were assessed by ultrasound at the carotid artery bifurcation, common carotid artery, and internal carotid artery on both sides. Plaques were defined as focal thickening of the vessel wall of at least 1.5 times the average intima-media thickness relative to adjacent segments with or without calcified components. The carotid artery plaque score (range 0–6) reflected the number of these locations with plaques. Information on blood pressure lowering medication use and education level (low: primary education, intermediate: secondary general or vocational education, or high: higher vocational education or university) was obtained during the home interview by a questionnaire. Definitions of a history of myocardial infarction, coronary artery bypass graft, and percutaneous coronary intervention, has been described extensively previously [15].

### Four-way decomposition method

**Fig. 2:**
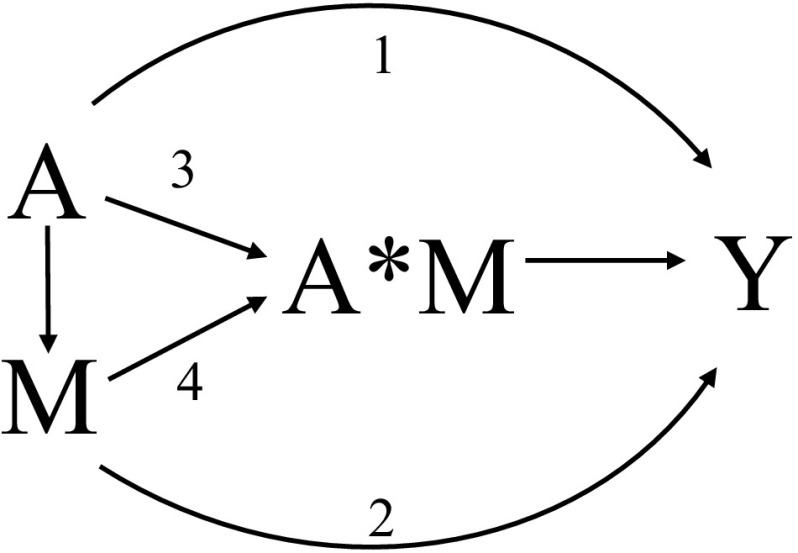
A drawing showing the four-way decomposition of the total effect of smoking (A) on incident stroke (Y) via wider venules (M). Interaction of smoking with wider venules is reflected by A*M. **Arrow 1** reflects the Controlled Direct Effect [CDE], effect of smoking (exposure) on stroke (outcome) independent of wider venules (mediator); **Arrow 2** reflects the pure indirect effect [PIE], the effect of smoking solely through wider venules without any interaction; **Arrow 3** reflects reference interaction [INT(ref)], the interaction between smoking and wider venules without smoking having an effect on wider venules; **Arrow 4** reflects mediated interaction [INT(med)], interaction between smoking and that component of wider venules, which is due to the effect of smoking

**Fig. 3:**
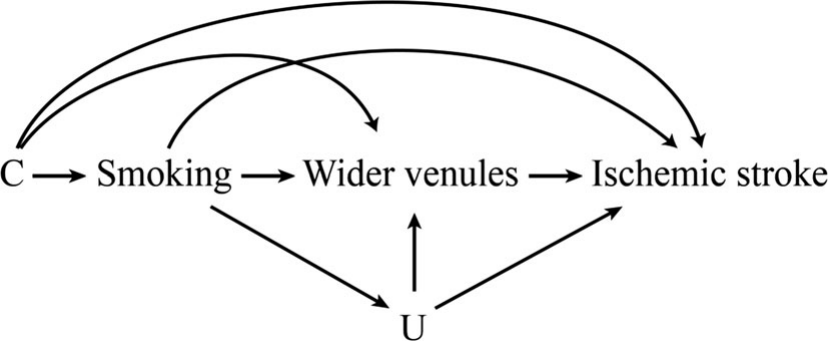
A causal diagram in which *C* confounds the smoking/ischemic stroke, wider venules/ischemic stroke, and smoking/wider venules relation. We assume there is no unmeasured confounding, and that smoking does not affect *C*. Here *C* and *U* refer to the following list of measured person characteristics: age, sex, subcohort, education, systolic blood pressure, diastolic blood pressure, blood pressure lowering medication use, body mass index, total cholesterol, high-density lipoprotein cholesterol, white blood cell counts, diabetes mellitus, alcohol intake, carotid plaques, history of cardiovascular disease, and retinal arteriolar caliber

In causal mediation analysis, the total causal effect of the exposure on the outcome can be decomposed into four components in the presence of an exposure-mediator interaction [10]: the controlled direct effect due to neither mediation nor interaction (in the current study: the effect of smoking on ischemic stroke which does not go through wider venules), reference interaction effect due to interaction alone (in the current study: the interaction of smoking with the observed level of wider venules), mediated interaction effect due to both mediation and interaction (in the current study: the interaction of smoking with wider venules that has been explicitly caused by smoking), and the pure indirect effect due to mediation alone (in the current study: the effect of smoking on ischemic stroke which purely goes through wider venules). Of note, for mediated interaction it is defined by the mediator having an effect on the outcome only in the presence of the exposure, but not in the absence of exposure, i.e. the exposure is necessarily present for the mediator to affect the outcome. Pure mediation is defined by the mediator having an effect on the outcome even in the absence of the exposure. Moreover, the controlled direct effect and reference interaction effect can be combined into the natural direct effect. Similarly, the mediated interaction effect and pure indirect effect can be combined into the natural indirect effect [16–18]. Figure 2 shows a conceptual drawing of the four-way decomposition, and Fig. 3 shows a causal diagram representing the association of smoking with risk of ischemic stroke mediated by wider venules. Several conditions need to be met in order to identify each of the four components, including the following assumptions concerning confounding: (1) the effect of smoking on ischemic stroke should be unconfounded conditional on the baseline covariates; (2) the effect of wider venules on incident ischemic stroke should be unconfounded conditional on smoking and baseline covariates; (3) the effect of smoking on wider venules should be unconfounded conditional on baseline covariates; (4) the mediator-outcome confounders should not be affected by smoking. Of note, for the total effect only the first of these assumptions is required; the controlled direct effect only requires the first and second assumptions [10].

### Statistical analyses

To obtain estimates of the four components from the data, a regression-based approach was used. First, we used a logistic regression model to regress incident ischemic stroke on smoking, retinal venular caliber, a product term denoting the interaction between smoking and the retinal venular caliber, and the covariates. Next, we used a linear regression model to regress retinal venular caliber on smoking and the covariates. We adjusted both regressions for the following covariates: age, sex, subcohort, education, systolic blood pressure, diastolic blood pressure, blood pressure lowering medication use, body mass index, total cholesterol, high-density lipoprotein cholesterol, white blood cell count, diabetes mellitus, alcohol intake, carotid plaque, history of cardiovascular disease, and the retinal arteriolar caliber. We obtained estimates of the four components by combining parameters from these two models according to the analytic expressions provided by VanderWeele [10]. Confidence intervals for the effect estimates were obtained via case resampling bootstrapping with 1000 iterations. We defined the proportion of the effect that was attributable to each component by dividing the estimate of a component by the excess relative risk. The overall proportion mediated was defined as the pure indirect effect plus mediated interaction divided by the excess relative risk; the overall proportion attributable to interaction was defined as the reference interaction plus mediated interaction divided by the excess relative risk; the overall proportion eliminated was defined as the excess relative risk minus the controlled direct effect divided by the excess relative risk.

In additional analyses, we aimed to address whether it is the smoking at the moment of the venular caliber assessment or the history of smoking that mattered most. Therefore, we redid the analyses by comparing ever smoking (i.e. past smoking and current smoking) to never smoking.

Finally, we performed sensitivity analyses to determine how much the estimates of the natural direct and indirect effect would change under different degrees of confounding by a hypothetical unmeasured binary confounder. Although we have adjusted for several potential confounders, the estimates will of course be biased if there remains important unmeasured or residual confounding of the relation between wider venules and ischemic stroke; here, for example, we may be concerned there is residual confounding by unhealthy lifestyle (e.g. unhealthy diets or physical inactivity). For this analysis, we used bias formulas for odds ratios for natural direct and indirect effects described in VanderWeele [19]. Briefly, bias-corrected-effect estimates for the natural direct and indirect effect were calculated by specifying a range of plausible values for the effect of the unmeasured confounder on ischemic stroke, and specifying a range of plausible values for the prevalence of unmeasured confounder among non-smokers and smokers. Next, these bias-corrected-effect estimates were subtracted from the crude natural direct and indirect effect (i.e. adjusted for age, sex, and subcohort) to address change in effect estimate if we had been able to adjust for this unmeasured confounder. Hence, a positive difference could be interpreted as an underestimation of the observed natural direct or indirect effect estimate failing to adjust for the unmeasured confounder, whereas a negative difference could be interpreted as an overestimation. All analyses were performed in R version 3.2.3.

## Results

Table 1 shows the baseline characteristics of the study population by gradable and ungradable retinal photographs. Compared to persons with gradable retinal photographs, persons with ungradable retinal photographs were older, were lower educated, were more users of blood pressure lowering medication, had a higher diastolic blood pressure, a higher serum total cholesterol, a lower serum high-density lipoprotein cholesterol, a higher white blood cell count, a higher daily alcohol intake, and had more often carotid plaques, diabetes mellitus, and a history of cardiovascular disease.

**Table 1:** Characteristics of the study population by gradable and ungradable retinal photographs

Characteristic	Gradable	Ungradable
N	9109	3611
Age (years)	63.7 (9.0)	71.7 (12.7)
Female sex (%)	5286 (58)	2251 (62)
Education (%)		
Low	3869 (43)	2020 (56)
Intermediate	3839 (42)	1191 (33)
High	1401 (15)	402 (11)
Systolic blood pressure (mmHg)	136.7 (21.0)	140.8 (22.5)
Diastolic blood pressure (mmHg)	77.2 (11.9)	76.4 (12.8)
Blood pressure lowering medication (%)	2638 (29)	1377 (38)
Body mass index (kg/m^2^)	26.9 (4.1)	26.7 (4.2)
Total cholesterol (mmol/l)	6.2 (1.3)	6.2 (1.3)
High-density lipoprotein cholesterol (mmol/l)	1.4 (0.4)	1.3 (0.4)
White blood cell (count/mm^3^)	6.7 (1.9)	6.9 (2.3)
Diabetes mellitus type 2 (%)	663 (7)	331 (9)
Daily alcohol intake (%)		
Nondrinker	2626 (29)	636 (18)
≤ 15 g	4251 (47)	1899 (53)
> 15 g	2232 (25)	1078 (30)
Carotid plaque score ≥ 4 (%)	1338 (15)	702 (19)
History of cardiovascular disease (%)	575 (6)	230 (6)
Smoking status (%)		
Non smoker	2921 (32)	1279 (35)
Past smoker	4012 (44)	1204 (33)
Current smoker	2176 (24)	742 (21)
Venular caliber (µm)	228.6 (23.1)	NA
Arteriolar caliber (µm)	150.8 (15.7)	NA

Values are presented as means (standard deviation) or as percentages

*NA* not applicable

Table 2 shows the baseline characteristics of the study population by smoking status. While some characteristics were similar across the groups, others were notably different. For example, comparing the groups to each other, never smokers were more often women and had a higher systolic blood pressure; past smokers had more often diabetes mellitus and a history of cardiovascular disease; current smokers had larger venular calibers and had more incident stroke cases.

**Table 2:** Characteristics of the study population by smoking status

Characteristic	Never smoking	Past smoking	Current smoking
N	2921	4012	2176
Age (years)	65.0 (10.0)	63.9 (8.5)	61.9 (8.2)
Female sex (%)	2397 (82)	1824 (46)	1065 (49)
Education (%)			
Low	1422 (49)	1494 (37)	953 (44)
Intermediate	1094 (38)	1823 (45)	922 (42)
High	405 (14)	695 (17)	301 (14)
Systolic blood pressure (mmHg)	138.3 (21.2)	136.9 (20.5)	134.2 (21.2)
Diastolic blood pressure (mmHg)	77.1 (11.8)	77.6 (11.8)	76.3 (12.2)
Blood pressure lowering medication (%)	886 (30)	1256 (31)	496 (23)
Body mass index (kg/m^2^)	27.1 (4.1)	27.1 (4.0)	26.1 (4.1)
Total cholesterol (mmol/l)	6.3 (1.3)	6.2 (1.2)	6.2 (1.3)
High-density lipoprotein cholesterol (mmol/l)	1.5 (0.4)	1.4 (0.4)	1.3 (0.4)
White blood cell (count/mm^3^)	6.3 (1.6)	6.5 (1.7)	7.8 (2.1)
Diabetes mellitus type 2 (%)	202 (7)	304 (8)	157 (7)
Daily alcohol intake (%)			
Nondrinker	1198 (41)	918 (23)	510 (23)
≤ 15 g	1412 (48)	1944 (49)	895 (41)
> 15 g	311 (11)	1150 (29)	771 (35)
Carotid plaque score ≥ 4 (%)	285 (10)	615 (15)	438 (20)
History of cardiovascular disease (%)	104 (4)	359 (9)	112 (5)
Venular caliber (µm)	223.8 (22.7)	227.7 (22.0)	236.8 (23.4)
Arteriolar caliber (µm)	149.4 (15.7)	149.9 (15.5)	154.7 (15.8)
Incident stroke cases	188 (6)	287 (7)	190 (9)

Values are presented as means (standard deviation) or as numbers (percentage)

During a mean follow-up of 12.5 years (113885.0 person-years), 665 persons had an ischemic stroke. Without adjustment for retinal vascular calibers, smoking increases the risk of ischemic stroke, odds ratio (OR): 1.38 (95% CI 1.13; 1.67), but this risk decreases to 1.34 (1.09; 1.63) after adjusting for the retinal vascular calibers.

Table 3 shows the results of the four-way decomposition. In the model accounting for the presence of an interaction between smoking and venules (*P* value for interaction = 0.029), smoking increases the risk of ischemic stroke: 1.41 (1.10; 1.67), whereas in the model accounting for the presence of an interaction between smoking and arterioles (*P* value for interaction = 0.737), the risk of smoking on ischemic stroke is 1.34 (1.06; 1.59). With venules as being the mediator, 77% of the excess relative risk of smoking on ischemic stroke compared to non-smoking is attributable to the controlled direct effect, 4% to only interaction, 15% to both interaction and mediation, and 4% is only attributable to mediation. Hence, the overall proportion of the effect of smoking on ischemic stroke that is mediated by wider venules is 19%. Redoing this causal mediation analysis but now comparing ever smoking to never smoking showed that 93% of the excess relative risk of ever smoking on ischemic stroke compared to never smoking is attributable to the controlled direct effect, whereas 11% to both interaction and mediation (*P* value for interaction = 0.004).

**Table 3:** Four-way decomposition of the effect of smoking on stroke by venules

	Current smokers compared to non-smokers
	Estimate (95% CI)
Total effect	1.41 (1.10; 1.67)
Excess relative risk due to each component	
Controlled direct effect	0.32 (0.03; 0.58)
Reference interaction	0.02 (− 0.03; 0.03)
Mediated interaction	0.06 (− 0.00; 0.11)
Pure indirect effect	0.02 (− 0.01; 0.04)
Proportion of effect due to each component	
Controlled direct effect	77% (60%; 120%)
Reference interaction	4% (− 10%; 8%)
Mediated interaction	15% (− 10%; 28%)
Pure indirect effect	4% (− 8%; 11%)
Overall proportion	
Mediated	19% (− 12%; 31%)
Attributable to interaction	19% (− 18%; 35%)
Eliminated	22% (− 20%; 39%)

Values are adjusted for age, sex, subcohort, education, systolic blood pressure, diastolic blood pressure, blood pressure lowering medication use, body mass index, total cholesterol, high-density lipoprotein cholesterol, white blood cell counts, diabetes mellitus, alcohol intake, carotid plaques, history of cardiovascular disease, and retinal arteriolar caliber

**Fig. 4:**
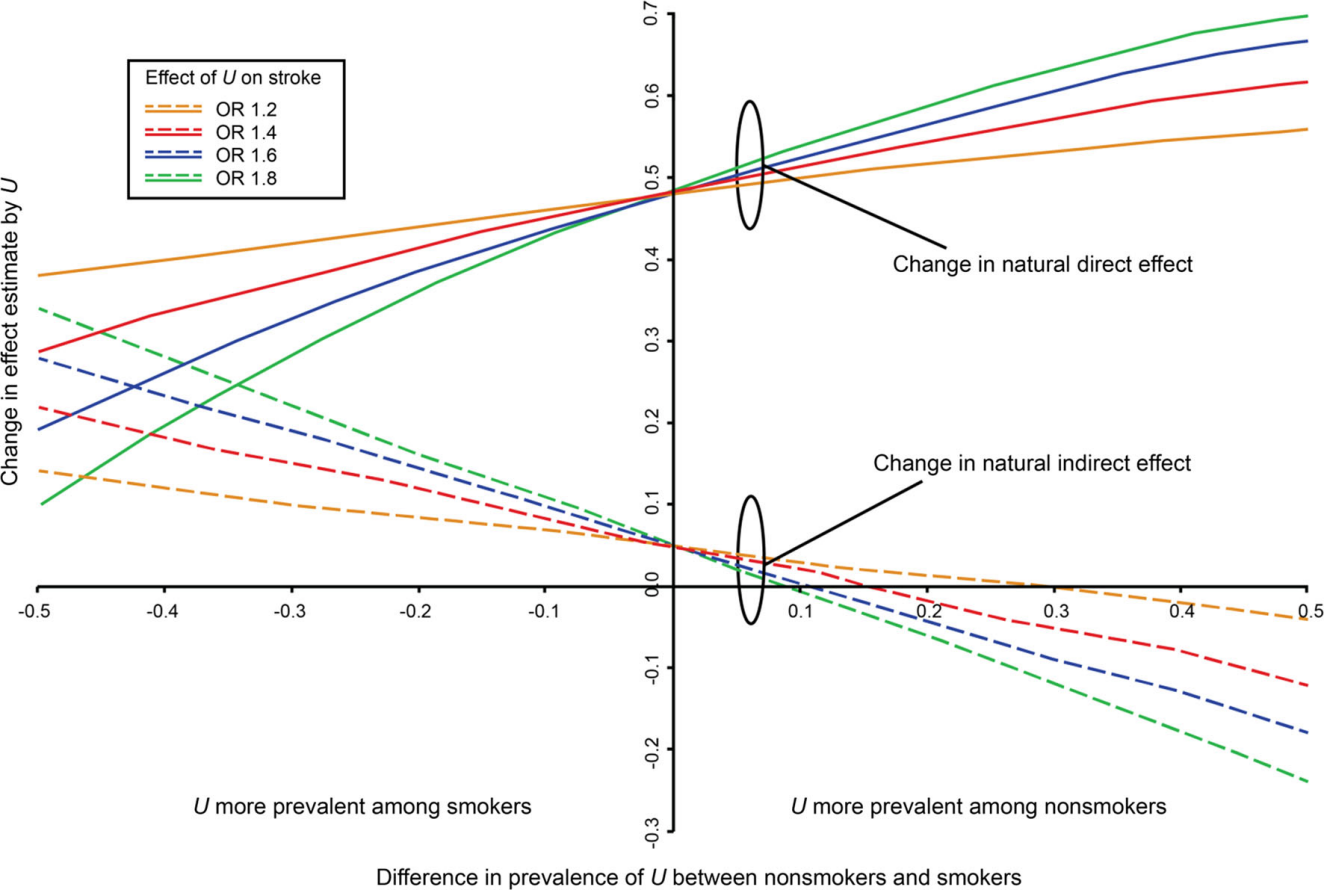
Illustrates how a crude natural direct effect estimate of 1.48 and a crude natural indirect effect estimate of 1.05 (the intercept on y-axis) would change under different magnitudes of confounding by a binary unmeasured variable *U*. The difference between the observed effect and bias-corrected-effect is depicted on the Y-axis, and the difference in prevalence of *U* between non-smokers and smokers is depicted on the X-axis. The colored lines are a range of plausible values for the effect of *U* on ischemic stroke. (Color figure online)

Figure 3 shows the results of the sensitivity analysis. Assuming that the unmeasured confounder (e.g. unhealthy lifestyle) increases the risk of ischemic stroke, and that it is more prevalent among smokers compared to non-smokers, the crude natural direct effect of 1.48 seems to overestimate the bias-corrected effect; the crude natural indirect effect of 1.05 seems to underestimate the bias-corrected effect. In these scenarios, the degree of mediation of smoking by venules would be underestimated without adjustment. In contrast, if the unmeasured confounder is more prevalent among non-smokers compared to smokers, the observed natural direct effect underestimates the true effect, whereas the natural indirect effect overestimates the true effect (Fig. 4).

## Discussion

In this population-based cohort study, we found that the effect of smoking on incident ischemic stroke is partly explained through changes in the venules, where there is both mediation and mediated interaction between smoking and the venules.

Thus far, studies focusing on the relation of microvascular damage with the risk of ischemic stroke found that wider venules, but not narrower arterioles were related to incident ischemic stroke [20, 21]. Given that smoking is related to wider venules and not to narrower arterioles, these findings suggest that the effects of smoking on ischemic stroke might be mediated by wider venules. Indeed, our indirect effect estimate (1.08, 95% CI 1.01; 1.14) supports this hypothesis. Importantly, we have further decomposed the direct effect into the controlled direct effect and reference interaction effect, and the indirect effect into the mediated interaction effect and the pure indirect effect, and indeed found evidence that the mediated component reflects in part mediation only and in part a mediated interaction between smoking and wider venules.

It is well-established that the components of the total effect decomposition we estimated here (with the exception of the controlled direct effect) do not directly address questions pertaining clinical or public health decision-making [22, 23]. Rather, this total effect decomposition can be seen as a means of exploring *why* certain total effects are present (in our study: why smoking affects ischemic stroke), and therefore can possibly inspire further research directions that lend themselves to more directly clinical implications. For example, a recent study has conceptualized mediated interaction and illustrated the concept of reversible and irreversible damage.^17^ This conceptualization of our mediated interaction effect estimate could be viewed as the effect of smoking on ischemic stroke by wider venules that is reversible. Similarly, the conceptualization of our pure indirect effect would be the effect of smoking on ischemic stroke by wider venules that is irreversible.^17^ With this particular interpretation, our findings suggest that smoking cessation might not lower the risk of ischemic stroke completely by changing venules. Practically, this could mean that once the venules have become wider due to smoking, even if you stop smoking, wider venules will affect the risk of ischemic stroke. Note, however, that explicitly assessing this hypothesis is feasible with longitudinal data on smoking behaviors and the venules. In other words, our findings inspire and support this as a topic for future research. Interestingly, one Japanese study has investigated the effect of smoking cessation on the retinal vascular calibers, and showed that the effect of smoking on wider venules is reversible in women, following 10 or more years of smoking cessation [24].

Several potential mechanisms may explain how the effect of smoking on stroke is partly attributable to wider venules. First and foremost, that smoking is associated with cardiovascular risk factors shared between venular calibers and ischemic stroke might explain the effect of smoking on ischemic stroke by wider venules. Although we have adjusted for these cardiovascular risk factors, there is still room for residual confounding which may partly explain our findings. Further, it is very likely that there is interaction between these shared cardiovascular risk factors which might contribute differentially to the development of ischemic stroke.

Apart from cardiovascular risk factors shared between venules and stroke, it is possible that smoking leads to a disturbance in venular efflux rather than arteriolar influx, and thereby causing stroke. Venous collagenosis i.e. thickening of periventricular veins and venules, has been observed with normal aging, and has been associated with increased venous pressure, venular dilatation and venular blood–brain barrier disruption. These alterations may cause white matter lesions and thereby play a role in ischemic stroke development. Also, other processes that are not related to smoking may lead to disturbances in venular efflux, and thereby strengthen the effect of smoking on ischemic stroke.

While increasing evidence suggest white matter lesions to be caused by venular changes, there is no evidence that other imaging markers of cerebral small vessel disease including lacunar infarcts and cerebral microbleeds are associated with venules. Hence, the effect of smoking on ischemic stroke may be mediated by white matter lesions caused by venular changes, and not by other imaging markers of cerebral small vessel disease. It might be that other cardiovascular risk factors such as high blood pressure may cause lacunar infarcts and cerebral microbleeds, and thus, studying such risk factors using causal mediation analysis may provide further insight into the effects of smoking on stroke.

Some limitations merit attention. Within the causal inference literature on mediation and interaction, it remains unclear to what extent each of the four components are susceptible to different forms of bias [10]. Recent evidence suggests interactions may be more robust to confounding, but may be more sensitive to measurement error when the two exposures are correlated [25]. Here, we reflect separately on confounding, selection bias, and information bias. First, it should be noted that the mediation analyses are subject to strong ‘no unmeasured confounding’ conditions [26]. As the Rotterdam Study is conducted in an ethnically homogenous Dutch population, it is unlikely that ethnicity related genes will result in major confounding of the smoking/wider venules, or smoking/ischemic stroke relation. Although we adjusted for many measured demographic and health-related person characteristics, residual or unmeasured confounding from socioeconomic status, sedentary lifestyle, physical inactivity, and unhealthy diets may affect our estimates. Currently, there are no bias formulas for each of the four components, and therefore, we performed a sensitivity analysis developed for the natural direct and indirect effects [19]. Given the expected direction of residual confounding (i.e. unmeasured confounders like unhealthy lifestyle increases the risk of ischemic stroke, and that smokers have more often an unhealthy lifestyle than non-smokers), our sensitivity analysis suggest that the indirect effects reported here may underestimate the true mediated effect while the direct effects are overestimated.

Concerning selection bias, persons without gradable retinal photographs excluded from our analyses had a worse cardiovascular risk profile, suggesting a possible but likely limited role for bias due to selecting on mediator assessments. Likely, these persons did not participate in our study due to their poor health condition which implicates the inclusion of relatively healthy persons in our study.

Ignoring measurement error have been suggested to overestimate the direct effect of an exposure, and thus, underestimates the indirect effect [27]. In our study, we did not use a dynamic measure of the venules, synchronized on the cardiac cycle, but a static measure. As such, this may have caused independent nondifferential misclassification leading to an underestimation of the indirect effects. Another independent nondifferential misclassification that may have occurred is due to the fact that we have used the retinal microvasculature as a proxy for the condition of the cerebral microvasculature. Given that the retinal and cerebral microvasculature have similarities in anatomy, physiology and embryology, the retinal microvasculature has been widely used to study vascular brain diseases [28]. However, it is conceivable that such proxy measures may have caused some independent nondifferential misclassification of the effect estimates. Also, it should be noted that the four-way decomposition method requires a relatively large sample size due to its decomposition of the effects. Hence, particular attention should be given to the strength of evidence as measured by the *P* value or confidence intervals.

In conclusion, we found that in the pathophysiology of ischemic stroke, the effect of smoking on the risk of ischemic stroke may partly be explained by changes in the venules, where there is both pure mediation and mediated interaction between smoking status and the venules. Our study shows that causal mediation analysis in clinical research may provide insights into the pathophysiology of clinical outcomes.
